# Comparative Study of Chemical Composition and Antioxidant Activity of Essential Oils and Crude Extracts of Four Characteristic *Zingiberaceae* Herbs

**DOI:** 10.3390/plants10030501

**Published:** 2021-03-08

**Authors:** Milena Ivanović, Kaja Makoter, Maša Islamčević Razboršek

**Affiliations:** Faculty of Chemistry and Chemical Engineering, University of Maribor, Smetanova ulica 17, SI-2000 Maribor, Slovenia; milena.ivanovic@um.si (M.I.); kaja.makoter@student.um.si (K.M.)

**Keywords:** *Zingiberaceae* family, cardamom, turmeric, galangal, ginger, essential oils, GC-MS/MS, antioxidant activity

## Abstract

The ginger family (*Zingiberaceae*) includes plants that are known worldwide to have a distinctive smell and taste, which are often used as spices in the kitchen, but also in various industries (pharmaceutical, medical, and cosmetic) due to their proven biological activity. The aim of this study was to investigate and compare the chemical composition and antioxidant activity (AA) of essential oils (EOs) of four characteristic ginger species: *Elettaria cardamomum* L. Maton (cardamom), *Curcuma Longa* L. (turmeric), *Zingiber Officinale* Roscoe (ginger), and *Alpinia Officinarum* Hance (galangal). Furthermore, the total phenolic content (TPC) and AA of crude extracts obtained after using ultrasound-assisted extraction (UAE) and different extraction solvents (80% ethanol, 80% methanol and water) were evaluated. A total of 87 different chemical components were determined by GC-MS/MS in the EOs obtained after hydrodistillation, 14 of which were identified in varying amounts in all EOs. The major compounds found in cardamom, turmeric, ginger, and galangal were α-terpinyl acetate (40.70%), β-turmerone (25.77%), α-zingiberene (22.69%) and 1,8-cineol (42.71%), respectively. In general, 80% ethanol was found to be the most effective extracting solvent for the bioactivities of the investigated species from the *Zingiberaceae* family. Among the crude extracts, ethanolic extract of galangal showed the highest TPC value (63.01 ± 1.06 mg GA g^−1^ DW), while the lowest TPC content was found in cardamom water extract (1.04 ± 0.29 mg GA g^−1^ DW). The AA evaluated by two different assays (ferric-reducing antioxidant power-FRAP and the scavenging activity of the cationic ABTS radical) proved that galangal rhizome is the plant with the highest antioxidant potential. In addition, no statistical difference was found between the AA of turmeric and ginger extracts, while cardamom rhizome was again inferior. In contrast to the crude extracts, the EOs resulted in significantly lower ABTS and FRAP values, with turmeric EO showing the highest AA.

## 1. Introduction

The ginger family (*Zingiberaceae*) consists of 53 genera and about 1300 different species, mainly distributed in South and South-East Asia [1]. Many herbs from this family have found applications in various industries (food, cosmetics, perfumery, pharmacy, etc.) due to their characteristic organoleptic properties (color, taste, odor) and their diverse chemical composition [2]. Indeed, *Zingiberaceae* species are a rich source of various phytochemicals, from alkaloids, carbohydrates, proteins, phenolic acids, flavonoids, and diarylheptanoids [3,4]. In addition, ginger plants are frequently used for the production of essential oils (EOs), which are typically rich in monoterpenes and sesquiterpenes [3,5]. Besides their well-known use in cosmetics, cleaning products, perfumes, and aromatherapy, EOs also serve as natural preservatives due to their proven antimicrobial and antifungal properties [6,7,8]. However, a thorough evaluation of the chemical profile of EOs is of great importance to uncover potential synergistic antimicrobial activities between EO compounds, as already shown for thyme [9,10] and ginger EO [11].

Cardamom (*Elettaria cardamomum* L. Maton) is a spice with a characteristic rich taste and aroma, also known as “true cardamom” or “green cardamom” [3], rich in essential oils, fatty acids, pigments, proteins, cellulose, sugars, starch, silica, calcium oxalate, and minerals [12]. The content of EOs in cardamom fruits varies between 0.2–6.2%, while the most dominant compounds found are 1,8-cineol (20–60%) and α-terpinyl acetate (20–55%) [3]. Based on the results published so far, the cardamom fruit has potential applications as an antimicrobial, antibacterial, antioxidant, and an efficient skin-permeation agent for certain drugs [13,14], but also as bacterial inhibitor [15].

The main compounds found in the rhizome of turmeric (*Curcuma longa* L., curcuma) are essential oil [16,17] and curcuminoids (including curcumin, dimetoxycurcumin, and *bis*-dimetoxycurcumin), a class of diarylheptanoids responsible for the characteristic orange-yellow color of turmeric spices, but also for their pharmaceutical properties [18]. In general, the therapeutic properties of curcuma include insecticidal, antimicrobial, antifungal, and antioxidant activity [16]. However, the results of published studies have also shown neuroprotective, hepatoprotective, cardiovascular, anti-inflammatory, antidiabetic, and anticancer effects of the turmeric extract [19,20,21].

The main ingredients of the ginger rhizome (*Zingiber officinale* Roscoe) are gingerols/shogaols and essential oil. Gingerols (characteristic compounds of the *Zingiberaceae* family, especially ginger) are the biologically main active components of fresh ginger, while gingerol derivatives, shogaols (dehydrated form of gingerols) are components of dried or cooked ginger [22]. Ginger is also rich in vitamins A and C, while the other compounds such as fatty acids, proteins, carbohydrates, fiber, ash, minerals (potassium, calcium, phosphorus, magnesium, iron) are present in lower amounts [23]. Recent studies have shown various pharmaceutical effects of the ginger rhizome, and the most important is the proven antidiabetic effect [24,25].

*Alpinia officinarum* Hance, commonly known as lesser galangal, with proven anti-inflammatory, cytotoxic, thermostabilizing, lipid regulating, antioxidant, antiviral, and antimicrobial properties, is also a rich source of phenolic compounds, in particular diarylheptanoids and essential oil [26]. However, despite its proven healing effects, some research has shown that diarylheptanoids are associated with some limitations including low oral absorption, bio-distribution, and systemic bioavailability, which lead to its failure in clinic as a drug [27].

In general, it can be stated that the antioxidant and pharmaceutical properties of *Zingiberaceae* plants are related to their chemical composition; this is primarily due to the presence of phenolic compounds and other biologically active constituents. Various extraction methods have already been evaluated for the isolation and purification of different bioactive compound classes from ginger spices, including classical hydrodistillation [2,5,28,29], extractions with supercritical fluids (supercritical CO_2_, supercritical water) [22,30,31], ultrasound-assisted extraction [32,33], and microwave-assisted extraction [34]. Gas chromatography-mass spectrometry (GC-MS) is a commonly used method for chemical characterization of EOs [1,29,32,35,36]. On the other hand, the polyphenolic profile of the selected herbs was usually evaluated by high performance liquid chromatography (HPLC) using different detectors [37,38,39]. In general, the chemical diversity of *Zingiberaceae* species and the content of the individual bioactivities have already been studied. For instance, based on the study reported by Elguindy et al., the major phenolics identified in the cardamom extract were tannic acid, gallic acid, caffeic acid, and 4,5-dicaffeoyl quinic acid [40]. In turmeric extracts, curcumin and its corresponding isomers, dimetoxycurcumin and bis-dimetoxycurcumin, were determined to be the major compounds in polar solvents [30]. The biological activity of ginger crude extracts is related to the presence of gingerol, turmeric, paradol, geraniol, geranial, borneol, linalool, camphene, zingerol, and zingiberone found in high concentrations [41], while Köse et al. in their study found flavonoids like kaempferol, apigenin, and luteolin as the most dominant compounds in galangal extracts [37].

However, to the best of our knowledge, there is no published work that systematically compares the chemical composition of the four selected *Zingiberaceae* herbs. Therefore, the main contribution of this work focuses on the study of the chemical composition and antioxidant activity (AA) evaluated by two different assay (ABTS and FRAP) of the essential oils of four plants, namely galangal, cardamom, turmeric, and ginger, which were extensively investigated after conventional hydrodistillation. In addition, the total phenolic content (TPC) and AA of the crude extracts were also examined. For this purpose, the plant material was processed by UAE using three different extraction solvents: 80% ethanol, 80% methanol, and water.

## 2. Results and Discussion

### 2.1. Essential Oils (EOs) Composition

The content of total extracted EOs in the selected species from the *Zingiberaceae* family varied from 0.29% to 3.74% of dry weight. GC-MS/MS analysis was used for the detailed analysis of EOs of the four selected plant species (cardamom fruit and rhizomes of turmeric, ginger and galangal) obtained by hydrodistillation. Consequently, GC-MS/MS analysis revealed the presence of a total of 87 different chemical structures (compounds), 14 of which were identified in varying amounts in all the EOs. All the identified compounds and their respective contents in the target plants are listed systematically in Table 1 in the order of elution from the non-polar VF-5ms capillary column.

**Figure 1:**
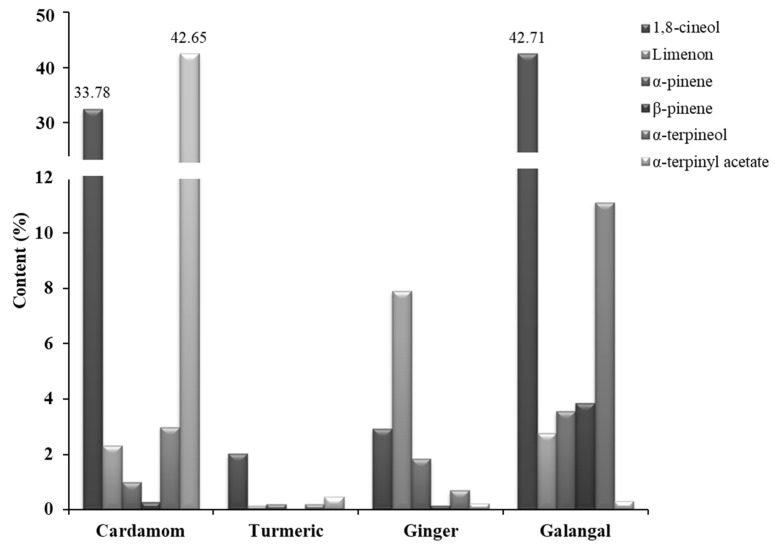
Comparison of the content of the most common chemical constituents found in the EOs of four selected *Zingiberaceae* species.

From these results (Table 1), it can be concluded that monoterpenes generally predominate in cardamom and galangal EOs, while sesquiterpenes are mainly found in turmeric and ginger EOs. Interestingly, cardamom rhizome contains the highest content of α-terpinyl acetate, which occurs in the low concentrations in the other herbs. The variations in the content of common individual compounds found in EOs of the *Zingiberaceae* species studied are shown in Figure 1.

The highest extraction yield of EO in this study was achieved with cardamom fruits originating from Guatemala (3.74%). Compared to the previously published works, this result was slightly higher. For example, Singh et al. achieved an extraction yield of 3.1% for the Indian samples [42], while Kuyumcu Savan and Küçükbay reported an extraction yield of 1% for the 3-h hydrodistillation of dried cardamom fruits from Turkey [43]. In our particular case, based on the GC-MS/MS analysis, a total of 22 chemical constituents were identified tentatively (Figure S1), which represents 99.15% of the total peak value recorded (Table 1). The most important compounds (99.04%) were monoterpenes, among which α-terpinyl acetate (42.65%) and 1,8-cineol/eucalyptol (33.78%) dominated. In addition to many proven pharmaceutical properties of cardamom EO (antioxidant, anti-inflammatory, antibacterial, anticancer, antifungal, and insecticidal effects) [3,14], the recently published study also showed that α-terpinyl acetate has multi-target directed ligand (MTDL) potential in Alzheimer’s disease [44]. The other compounds with a significant content in cardamom EO were α-terpineol (2.98%), linalool (2.72%), limonene (2.32%), 4-terpineol (1.85%), and sabinene (1.82%). These results are in good agreement with the previously published works for the cardamom samples from Guatemala [32,45,46].

The extraction yield for EO from curcuma rhizome was 0.25%, which is comparable with the results published by Naz et al., who achieved a yield of 0.67% for the Pakistani sample [47], but significantly lower than the result published by Zhang et al. (4.03%) for the samples from China [48]. This inconsistency in the results can be explained by differences in the origin of the sample, the time of harvest, the characteristics of the sample (dried or fresh samples), and the extraction method used [49]. Similarly, the plant material analyzed in this work represents the products available on the Slovenian market. Consequently, the process of storage, distribution, and preservation may influence the chemical composition and quality of the products themselves [50,51,52]. The most important of the 44 compounds identified in our specific sample were β-turmerone (25.77%) and ar-turmerone (12.28%), followed by ar-curcumen (11.42%), β-sesquiphellandrene (10.44%), curlone (7.59%), and α-zingiberene (5.13%) (Table 1 and Figure S2). The results are in good agreement with those presented in the previously published studies on the characterization of curcuma samples from China [2,16]. However, in the curcuma samples from India, the mainly confirmed volatile integrities were α-phellandrene, β-sesquiphellandrene, and 1,8-cineol [17]. Furthermore, the chemical composition of the curcuma leaves differs significantly from the chemical composition of the rhizome. In fact, EO of the curcuma leaves contains terpinol (52.88%) and α-phellandrene (21.13%) as the main constituents [53]. However, clinical studies have shown that turmerones (in particular ar-turmerone and β-turmerone) are the dominant compounds responsible for the pharmaceutical properties of curcuma EO, justifying greater use of the turmeric rhizome over the plant leaves [54]. Indeed, based on the studies published so far, anti-inflammatory [55], anti-invasion [55], anti-angiogenic [54], and anti-tumor [20] effects of turmerones have been reported.

In the case of the ginger rhizome originating from Madagascar, the yield of the extracted EO was 0.29%, and was comparable to the previously published results for the hydrodistillation. For example, Al-Dhahli et al. reported extraction yields of 0.14% and 0.20% for Chinese and Saudi ginger [36]. However, a significantly improved extraction yield (2.62%) was achieved by Mesomo et al., who used supercritical CO_2_ at 25.0 MPa and 333.15 K without reporting changes in oil quality [31]. The main components identified in our sample were α-zingiberene (22.69%), ar-curcumene (11.63%), β-sesquiphellandren (10.08%), limonene (7.90%) and β-bisabolene (7.55%) (Figure S3). The results are in good agreement with the recently published study by Camero et al. [56]. Several other studies have also confirmed α-zingiberene as the main ingredient of ginger EO from Ghana, Thailand, Poland, Nigeria, Australia and India [36,57,58], while ar-curcumene has been identified as the main ingredient of Brazilian and Cuban genotypes [59]. From a pharmaceutical point of view, ginger extracts represent a potentially effective preventive agent against various carcinogenic cells [60].

Finally, the yield of EO extracted from the galangal rhizome was 0.35%, with no difference between the results published by Raina et al. [29]. Fifty-three compounds representing 89.69% of the total oil were tentatively identified by GC-MS/MS and are listed in Table 1. The main compounds identified were 1,8-cineol (42.71%), α-terpineol (11.11%), followed by camphene (4.58%), β-pinene (3.85%), α-pinene (3.56%), limonene (2.76%), 4-terpineol (2.29%), and camphor (2.24%) (Figure S4). The occurrence of 1,8-cineol as the main component of the rhizome oil of *A. galanga* in the present study is consistent with earlier published results [2,28,29]. 1,8-cineol, also known as eucalyptol, has demonstrated several clinical activities including therapeutic benefits in inflammatory airway diseases, such as asthma or chronic obstructive pulmonary disease [61], and it also possesses anti-inflammatory [62] and anti-oxidative [62] properties.

### 2.2. Total Phenolic Content (TPC) of the Crude Extracts

**Figure 2:**
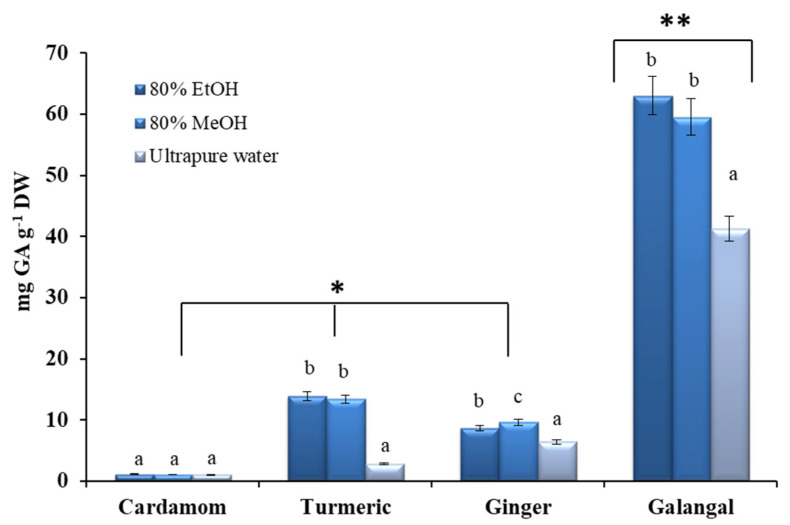
Total phenolic content (TPC) for crude extracts of the selected *Zingiberaceae* species, expressed as mg of gallic acid equivalents per gram of dry weight (mg GA g^−1^ DW). All measurements were performed in duplicate. For comparison of the mean values, one-way ANOVA followed by S-N-K post hoc was used. Different superscript letters indicate statistical differences in the extraction efficiency of the tested solvents (*p* < 0.05), while asterisks indicate statistical differences in TPC content between different plants (*p* < 0.05).

Figure 2 shows the total phenolic content (TPC), expressed as mg gallic acid equivalent per gram of dry weight (mg GA g^−1^ DW) for aq. MeOH (80% MeOH), aq. EtOH (80% EtOH) and water extracts of the selected *Zingiberaceae* species. In general, TPC values varied significantly between the plant samples, ranging from 1.13 mg GA g^−1^ DW to 63.00 mg GA g^−1^ DW. On the other hand, no statistically significant difference was generally found between the extraction efficiency of 80% MeOH and 80% EtOH for TPC. However, the extraction efficiency of ultrapure water was the lowest in all tests, except for the cardamom rhizome, where the same-very low TPC values were achieved in all evaluated solvents. The choice of the appropriate solvent from the point of view of the utilization of the extraction, but also its impact on the environment, is one of the most important factors in the selection of an extraction technique. Although some authors have highlighted methanol as the best solvent for the extraction of bioactive plant constituents [63,64], the use of so-called GRAS (generally recognized as safe) solvents such as water or aqueous ethanol solutions is a desirable alternative for the further use of the extracts obtained in the cosmetic, food and pharmaceutical industries [65,66]. Consequently, from the results presented in Figure 2, we can generally conclude that 80% EtOH represents a good extraction solvent for isolation of bioactive components from plant material of *Zingiberaceae* species.

Comparing the content of bioactive phenolic compounds expressed as TPC among the studied plants, the lowest TPC value was determined in cardamom rhizome and correlates well with previously published studies [24,33,38]. The low TPC content in cardamom can be explained by the fact that the flavonoid diosmin, the most important single phenolic compound [38], is practically insoluble in water and poorly soluble in polar organic solvents (e.g., EtOH, MeOH) [67].

In the case of the turmeric rhizome, the TPC of the water extract was 2.80 ± 0.19 mg GA g^−1^ DW, while the TPC values measured in the alcoholic extracts were significantly higher, namely 13.40 ± 0.66 mg GA g^−1^ DW and 13.93 ± 0.81 mg GA g^−1^ DW for 80% MeOH and 80% EtOH, respectively. Again, the results can be explained by the practical insolubility in water (<8 µg mL^−1^) of the main bioactive turmeric compounds (curcuminoids), and their good solubility in polar organic solvents, like methanol and ethanol [68]. However, our results for curcuma were somewhat higher compared to the recently published work of Yang et al. [69]. The disagreement in the results can be explained by differences in the origin of the samples, as we analyzed curcuma sample from India, while Yang et al. analyzed a Chinese sample [69].

In the case of the ginger rhizome, the highest TPC value (9.63 ± 0.05 mg GA g^−1^ DW) was found in the methanolic extract, while water resulted in the lowest TPC content (6.42 ± 0.33 mg GA g^−1^ DW). Similar results were observed in the study by Hester et al. [70].

Finally, the galangal extracts showed the highest TPC values without a statistically significant difference in the content between the 80% EtOH (63.00 ± 1.06 mg GA g^−1^ DW) and 80% MeOH (59.52 ± 4.75 mg GA g^−1^ DW). As previously reported, in addition to the volatile compounds identified in EO, the galangal rhizome is a rich source of various flavonoids, including kaempferol, apigenin, luteolin, quercetin, and isorhamnetin [37]. Our results for TPC (for the samples originating from China) are comparable to the results reported by Lu et al. [39].

### 2.3. Antioxidant Properties of Crude Extracts and Essential Oils of Zingiberaceae Species

In this study, two different in vitro tests, ferric reducing antioxidant capacity (FRAP) and ABTS radical scavenging activity, were also performed to evaluate the antioxidant properties of crude extracts and essential oils of selected plant material.

#### 2.3.1. FRAP Assay

FRAP assay of the crude extracts and essential oils was evaluated and the final results were expressed as mg Fe^2+^ per gram of dry weight (mg Fe^2+^ g^−1^ DW) and mg Fe^2+^ per milliliter of essential oils (mg Fe^2+^ mL^−1^ EO), respectively.

**Figure 3:**
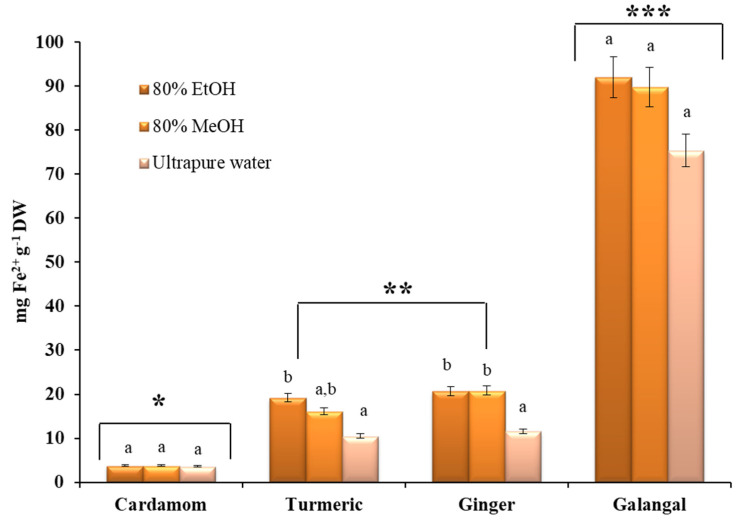
Antioxidant activity of selected *Zingiberaceae* species expressed as mg Fe^2+^ ion equivalents per gram of dry weight (mg Fe^2+^ g^−1^ DW). All measurements were performed in duplicate. For comparison of the mean values, one-way ANOVA followed by S-N-K post hoc were applied. A different superscript letters (a, b) indicate a statistical difference in the extraction efficiencies of the solvents tested (*p* < 0.05), while asterisks indicate statistical differences in antioxidant activity between different plants (*p* < 0.05).

In the case of crude extracts (Figure 3), alcoholic extracts have the highest antioxidant capacity, although for cardamom and galangal no statistically significant difference was found between the solvents tested. These results are in good agreement with the results published by Lu et al., who evaluated the antioxidant capacity of the 15 commonly used species from China [39].

As shown in Figure 3, the highest values (between 75.39 ± 10.57 mg Fe^2+^ g^−1^ DW and 92.02 ± 4.09 mg Fe^2+^ g^−1^ DW) were found for galangal. A similar trend was observed in the study by Köse et al., who evaluated the antioxidant activity of galangal extracts obtained with water, ethanol and water/ethanol mixture [37]. On the other hand, the lowest FRAP values were observed for cardamom fruits. Finally, no statistically significant difference was found between the reduction capacity of turmeric and ginger crude extracts (Figure 3). The highest FRAP value for turmeric was observed for ethanolic extract (20.82 ± 0.42 mg Fe^2+^ g^−1^ DW), which was comparable to the results published by Yang et al. [69]. In the case of the ginger rhizome, the same values were determined for ethanolic (20.73 ± 0.70 mg Fe^2+^ g^−1^ DW) and methanolic (20.82 ± 0.42 mg Fe^2+^ g^−1^ DW) extracts, while water was inferior as the extraction solvent with a FRAP value of 11.58 ± 1.26 mg Fe^2+^ g^−1^ DW.

**Figure 4:**
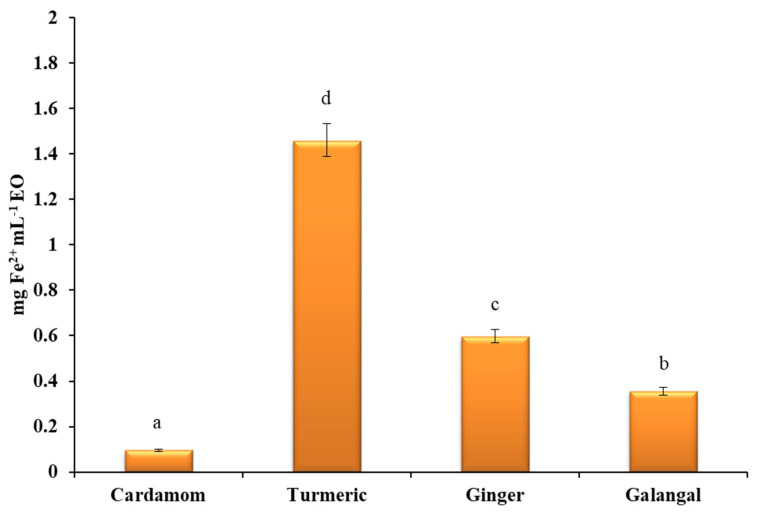
Antioxidant activity of EOs expressed as mg Fe^2+^ ion equivalents per milliliter of essential oil (mg Fe^2+^ mL^−1^ EO). All measurements were performed in duplicate. For comparison of the mean values, one-way ANOVA followed by S-N-K post hoc were applied. A different superscript letters (a, b, c, d) indicate a statistical difference in AA of evaluated EOs (*p* < 0.05).

The reducing power of the EOs (Figure 4) was again the lowest for the cardamom sample, while the highest FRAP value was obtained for the EO from turmeric. In contrast to the crude extracts, the EO from galangal showed weaker ferric reducing power. Ginger and galangal oils showed similar antioxidant activities as the study published by Avci et al. [71]. These results can probably be explained by differences in the chemical composition within the EOs studied. Namely, ginger EO and especially turmeric EO are rich in sesquiterpenes, whereas the dominant volatile compounds in cardamom and galangal were monoterpenes. Turmerones (especially ar-turmerone and β-turmerone) are the main compounds believed to be responsible for the antioxidant properties of turmeric EO [72]. For example, in the study of Gounder and Lingamallu, which demonstrated high antioxidant activity of turmeric EO, a significant correlation was found between the reduction of FRAP and the decrease of turmerone content [49]. A similar trend was also observed in the study published by Avanço et al. [16].

#### 2.3.2. ABTS Assay

In addition, ABTS assay was performed, where the ability of the crude extracts and EOs to scavenging the ABTS^•+^ was expressed as mg trolox equivalent per gram of dry weight (mg TE g^−1^ DW) or mg trolox equivalent per milliliter of essential oil (mg TE mL^−1^ EO), respectively.

**Figure 5:**
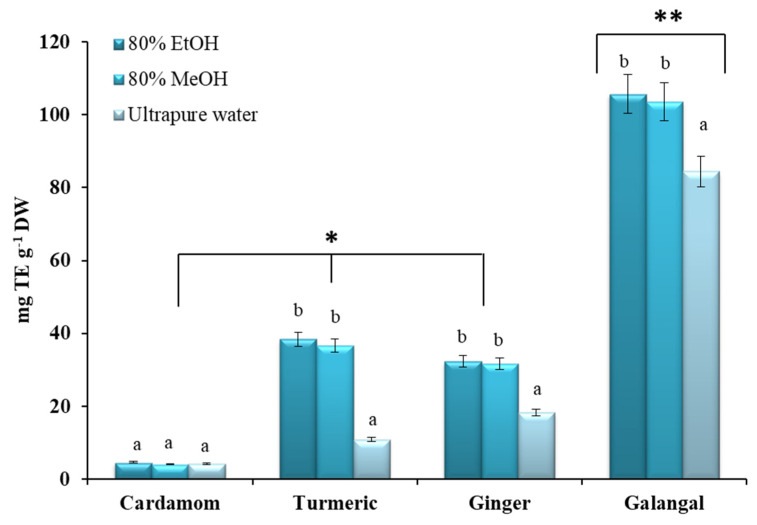
Antioxidant activity of selected *Zingiberaceae* species expressed as mg trolox equivalents per gram of dry weight (mg TE g^−1^ DW). All measurements were performed in duplicate. For comparison of the mean values, one-way ANOVA followed by S-N-K post hoc were applied. A different superscript letters (a, b) indicate a statistical difference in the extraction efficiencies of the solvents tested (*p* < 0.05), while asterisks indicate statistical differences in antioxidant activity between different plants (*p* < 0.05).

In the case of the crude extracts, it can generally be concluded that galangal possesses the highest ABTS^•+^ scavenging activity, while no significant differences were found between the other three plants (Figure 5). By comparison, Köse at al. showed that galangal has marked antioxidant, anticholinergic, reducing, radical scavenging, and metal binding activity [37].

In the case of cardamom rhizome, similar, very low results (ranging from 4.10 mg TE g^−1^ DW to 4.57 mg TE g^−1^ DW) were observed for all solvents tested (Figure 5). These results are in good agreement with results reported by Przygodzka et al. who classified cardamom in the group of species with low antioxidant activity [33]. Przygodzka et al. have also confirmed a statistically significant correlation between the TPC of the crude ethanolic extracts and the antioxidant ability of the studied plant material [33].

In contrast, no differences were found between 80% EtOH and 80% MeOH for turmeric, ginger and galangal, while water extracts showed the lowest values. Compared to the results published by Yang et al., for ABTS values for 80% EtOH turmeric extract, the results obtained in our study were slightly higher [69]. However, the study by Yang et al., confirmed a positive statistically significant difference in the extraction efficiency of UAE over the conventionally used solid/liquid extraction [69]. In general, our results are in good agreement with the results of Sana et al. [73], who investigated the effect of extraction parameters and extraction solvents on the antioxidant activity of turmeric.

Finally, in this study, the TPC values of the crude extracts of *Zingiberaceae* species obtained with the tested solvents (80% EtOH, 80% MeOH and water) were highly positively correlated with the AA values determined by ABTS and FRAP assays. The highest positive correlation (0.01 significant level) was observed for the water extracts with the correlation coefficients of r = 0.999 and 0.998 between TPC and ABTS and between TPC and FRAP, respectively. In the case of alcoholic extract, TPC was positively correlated with ABTS at the significant level of 0.05 (r = 0.985 and r = 0.988 for 80% EtOH and 80% MeOH, respectively), while the higher correlation (0.01 significant level) was found between TPC and FRAP values (r = 0.995 for ethanolic extracts and r = 0.994 for methanolic extracts).

**Figure 6:**
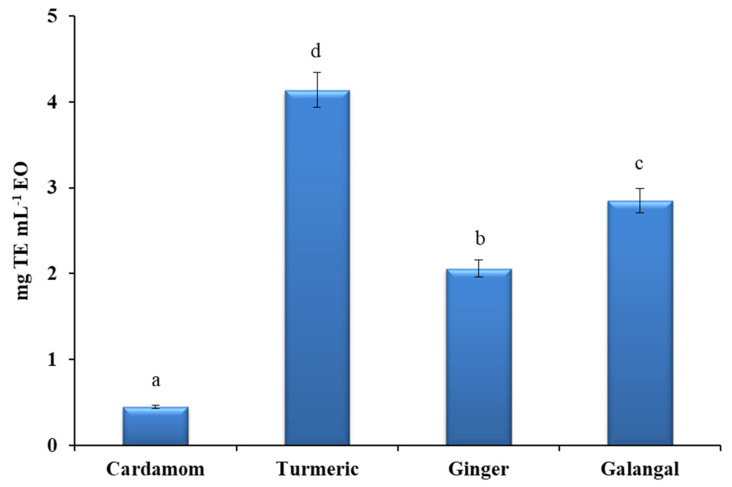
Antioxidant activity of EOs expressed as mg trolox equivalents per milliliter of essential oil (mg TE mL^−1^ EO). All measurements were performed in duplicate. For comparison of the mean values, one-way ANOVA followed by S-N-K post hoc were applied. Different superscript letters (a, b, c, d) indicate a statistical difference in AA of evaluated EOs (*p* < 0.05).

A statistically significant difference was found in the EOs among all the species studied (Figure 6). Compared to the crude extracts, the EOs generally showed weaker ABTS^•+^ scavenging activity. The highest ABTS value expressed as trolox equivalents (4.14 mg TE mL^−1^ EO) was observed for turmeric EO, followed by galangal, ginger, and finally cardamom EO.

## 3. Materials and Methods

### 3.1. Chemicals and Plant Material

Folin Ciocalteu’s reagent (FCR), anhydrous Na_2_CO_3_, FeSO_4_∙7H_2_O, acetic acid (99.8%) and gallic acid standard (99%) were supplied by Merck (Darmstadt, Germany). 2,4,6-*tris*(2-pyridyl)-*s*-triazine (TPTZ reagent, ≥99%), 2,2′-azino-*bis* (3-ethylbenzothiazoline-6-sulfonic acid) diammonium salt (ABTS reagent), (±)-6-hydroxy-2,5,7,8-tetramethylchromane-2-carboxylic acid (trolox, 97%), potassium persulfate (K_2_S_2_O_8_), FeCl_3_∙6H_2_O, sodium acetate (CH_3_COONa), anhydrous sodium sulfate (Na_2_SO_4_) and *n*-hexane (97%) were from Sigma Aldrich (St. Louis, MO, USA). HPLC-grade methanol (MeOH) and ethanol (EtOH, 96%) were supplied by Honeywell (Frankfurter, Germany), while ultrapure water was treated in the laboratory on a daily basis.

Dried plant material of selected *Zingiberaceae* species (cardamom, turmeric, ginger and galangal) was generously provided by the specialized market “Natural loti” Rakek, Slovenia. In accordance with the supplier specification, the plant material was dried in drying chambers at a controlled temperature (30 °C), with constant air circulation and while maintaining optimum humidity conditions. Prior to distribution, the dried material was stored in dry, airy, and cool areas, packed in tightly sealed containers to preserve flavor, color, and aroma and prevent access to moisture. The laboratory samples were ground in the electric blender (Profi Cook PC-KSW1021) for 1 min at room temperature, homogenized, and kept in a dark location before analysis. Information on the analyzed plant material is presented in Table 2.

### 3.2. Hydrodistillation of eEssential Oils from the Selected Species of Zingiberaceae Family

For the extraction of the essential oils (EOs) the classical hydrodistillation in the Clevenger apparatus was carried out [35]. Here, 100 g of the ground plant material was weighed and 600 mL ultrapure water was added. The heating temperature of the calotte was set to 130 °C, and the extraction was performed under these conditions for 3 h. The oils collected over anhydrous sodium sulfate were automatically weighed and analyzed. All extractions were carried out in duplicate.

### 3.3. GC and GC-MS/MS Analysis of the Essential Oils

For chemical identification and quantification, 1 µL of properly diluted EOs in *n*-hexane (1:20, *v:v*) was analyzed using a gas chromatograph Varian Saturn 2100T coupled to a MS/MS Saturn 2100 ion trap mass spectrometer. The chromatographic separation was performed with the capillary column VF-5ms (30 m × 0.25 mm × 0.25 μm). The split injection mode was used (split ratio 1:20), while He 6.0 was used as carrier gas with a flow rate of 1 mL min^−1^. The injector temperature was set to 230 °C. The initial oven temperature was set to 40 °C for 4 min, then the temperature was raised to 150 °C at a rate of 5 °C per min and held at this temperature for 13 min. In addition, the column was heated up to 200 °C at a rate of 10 °C and finally kept at 200 °C for the next 15 min. The total running time was 59 min. The mass spectra were recorded in the SCAN mode in a range from 50 to 650 *m/z* using electron ionization energy at 70 eV and the detector temperature was set to 150 °C. The volatile compounds were identified by comparing the mass spectra of the compounds in plant samples with those available in the NIST library and the data available in the literature.

### 3.4. Preparation of Crude Solvent Extracts by Ultrasound-Assisted Extraction (UAE)

For the evaluation of the content of non-volatile bioactive compounds, an UAE extraction was carried out. For this purpose, 500 mg of ground plant material was weighed into a 50 mL conical centrifuge tube and 10 mL of selected solvent (80% MeOH or 80% EtOH or ultrapure H_2_O) was added. The extractions were performed in an ultrasonic bath (Vevor, Shanghai, China) under the evaluated temperature (50 ± 1 °C) for 30 min. The extracts obtained were then centrifuged with a laboratory centrifuge (Eppendorf, 5804R, Hamburg, Germany) for 10 min at 10,000 rpm and the same extraction protocol was repeated once again with new 10 mL of the extraction solvent (80% MeOH or 80% EtOH or ultrapure H_2_O). The supernatants were combined in a 25 mL volumetric flask and made up to the mark with a suitable solvent. Before analysis, the extracts were filtered through a 0.45 µm PTFE filter and properly diluted (usually 1:5, *v:v*). All extractions and instrumental analysis were performed in duplicate.

### 3.5. Determination of Total Phenolic Content (TPC) of Crude Solvent Extracts

The total phenolic content (TPC) for the crude extracts of the selected *Zingiberaceae* species was determined by the standard spectrophotometric method first described by Singleton, with some modifications [74,75]. First, different concentrations (25, 50, 100, 150, 250, and 500 mg/L) of the standard solutions of gallic acid were prepared. After that, 3160 µL of ultrapure water was mixed with 40 µL of properly diluted extracts or gallic acid standard solutions and 200 µL of a 10% FC reagent. The mixture was left to stand for 6 min and 600 µL of a 20% Na_2_CO_3_ solution (*w:v*) was added. After 2 h of incubation at the dark place, the absorbances were measured at 765 nm against ultrapure water as a blank. The concentrations were expressed as mg gallic acid equivalent per gram of dry weight (mg GA g^−1^ DW).

### 3.6. Determination of Antioxidant Activity of Crude Extracts and Essential Oils

#### 3.6.1. ABTS Radical Scavenging Assay

The cationic ABTS^•+^ radical solution was prepared by mixing equal volumes of a 7 mM solution of ABTS (2,2′-azino-bis(3-ethylbenzothiazoline-6-sulfonic acid) diammonium salt) and a 2.4 mM solution of potassium persulfate (K_2_S_2_O_8_) and was incubated for 12–16 h in a dark place at room temperature (RT). Before use, this solution was further diluted with absolute EtOH to adjust the absorbance to 0.70 ± 0.05 at 734 nm (about 1 mL of ABTS^•+^ solution corresponds to 55–60 mL of EtOH) [76]. In addition, 3950 µL of the reaction solution was mixed with 50 µL of standard trolox solutions (in the concentration range of 0.1–1.0 mM) or properly diluted crude extracts or properly diluted essential oils. After a 30 min incubation in the dark at RT, the absorbances at 734 nm were measured against the blank solution (3950 µL ABTS^•+^ and 50 µL absolute EtOH). The scavenging effect, expressed in %, was calculated using the following equation:

(1) $$\mathrm{Scavenging}\;\mathrm{effect}\;\left(\%\right)=\;\frac{\left(\mathrm{AB}\;-\;\mathrm{AA}\right)}{\mathrm{AB}}\;\times \;100\;$$

where AB represents the absorbance of the mixture of ABTS^•+^ and EtOH (blank value), while AA represents the absorbance of the mixture of ABTS^•+^ and trolox solution/extract/EO. The final concentrations were expressed as mg trolox equivalent per gram of dry weight (mg TE g^−1^ DW) or mg trolox equivalent per milliliter of essential oil (mg TE mL^−1^ EO), respectively.

#### 3.6.2. Ferric Reducing Antioxidant Power Test (FRAP Assay)

The antioxidant activity of the crude extracts, expressed as mg Fe^2+^ ion equivalent per gram of dry weight (mg Fe^2+^ g^−1^ DW) and essential oils, expressed as mg Fe^2+^ ion equivalent per milliliter of essential oil (mg Fe^2+^ mL^−1^ EO), was determined by a standard spectrophotometric method with slight modifications [77]. In short, the daily fresh reagent FRAP was prepared by mixing acetate buffer (pH = 3.60), 10 mM TPTZ solution in 40 mM HCl, and 20 mM FeCl_3_ 6H_2_O in the volume ratio 10:1:1 at 37 °C [78]. Working solutions of Fe^2+^ ions in the concentration range 10–300 mg L^−1^ were prepared by diluting 20 mM Fe^2+^ standard solution with ultrapure water. For the measurements, 4950 µL of the reagent FRAP was mixed with 50 µL of properly diluted crude extracts/properly diluted essential oil or Fe^2+^ working solutions or properly diluted crude extract or properly diluted essential oil. After 30 min of incubation at 37 °C, the absorbances at 593 nm were measured against FRAP reagent as a blank value.

### 3.7. Statistical Analysis

All results were expressed as mean value ± standard deviation. The SPSS software: IBM SPSS Statistics for Windows, Version 22.0 (Armonk, NY: IBM Corp., published 2013) was used for the statistical evaluation of the results obtained. For that purpose, a one-way analysis of variance (ANOVA) at a 95% confidence level and a Student-Newman-Keuls (S-N-K) post-hoc test were applied. The correlation analysis between TPC and AA of the crude extracts of the selected spices was performed by calculating Pearson’s correlation coefficient.

## 4. Conclusions

Many plant species contain valuable EOs that are useful in many different areas, mainly because of their strong odor and because they have been shown to have a wide variety of pharmacological effects. The main constituents of EOs are monoterpenes, which have a pronounced antiseptic and antibacterial effects. In second place are the sesquiterpenes, which have anti-inflammatory and analgesic effects. In our study, we successfully isolated the EOs of four plant species belonging to the *Zingiberaceae* family, namely cardamom, turmeric, ginger, and galangal, and quantified their major constituents. The results confirmed that 1,8-cineol (2.01–42.71%), α-terpinyl acetate (0.21–42.65%), limonene (0.15–7.90%), α-pinene (0.20–3.56%), and β-pinene (0.03–3.85%) were the main monoterpenes in all the oils studied. Antioxidant assay (ABTS and FRAP) pointed out that turmeric EO possessed the highest antioxidant capacity, while the lowest value was determined for cardamom EO. In addition, the antioxidant activity of crude aqueous, ethanolic, and methanolic extracts was compared with different antioxidant assays and the results generally confirmed galangal extracts as those with the strongest antioxidant activity, followed by turmeric and ginger extracts, while cardamom extracts showed the lowest antioxidant capacity. Overall, the best extraction of antioxidant components was obtained when ethanol was used as a solvent followed by extraction with methanol and water. Many papers have been published on the antioxidant activity of *Zingiberaceae* species. However, the data show much inconsistency between the same essences or extracts, so direct comparison of results is very difficult. The reasons for this variability can be understood by considering all the factors that influence the chemical composition of the obtained extracts, namely climatic, seasonal, and geographical conditions, harvest period, parts of the plant used, distillation or extraction technique and antioxidant activity assay applied, among others. Anyhow, our results confirmed that plant species belonging to the family *Zingiberaceae* can serve as a good natural source of antioxidant components and natural EOs that can be widely used in modern pharmaceutical, food, nutraceutical, and cosmetic industries.

## Figures and Tables

**Table 1:** Chemical composition of essential oils (EOs) of four *Zingiberaceae* plants identified by GC-MS/MS after 3 h of hydrodistillation.

No.	Chemical Compound	Peak Area Percentage (%)				Classification
		Cardamom	Turmeric	Ginger	Galangal	
		*Elettaria cardamomum* L. Maton	*Curcuma longa* L.	*Zingiber officinale* Roscoe	*Alpinia officinarum* Hance	
**1**	4-acetyl-1-methyl-1-cyclohexene	-	0.03	-	-	other
**2**	*p*-acetyl toluene	-	0.08	-	-	other
**3**	trans-anethole	-	0.04	-	0.42	other
**4**	*cis*-α-bergamotene	-	-	0.11	-	sesquiterpene
**5**	*trans*-α-bergamotene	-	0.09	0.08	0.22	sesquiterpene
**6**	Borneol	-	-	0.04	0.06	monoterpene
**7**	β-bisabolene	-	3.04	7.55	0.38	sesquiterpene
**8**	Butyl isobutyrate	-	-	-	0.34	other
**9**	sec-butyl isobutyrate	-	-	-	0.19	other
**10**	Benzyl acetone	-	-	-	0.14	other
**11**	3-carene	-	0.03	-	-	monoterpene
**12**	o-cymene	-	0.01	-	-	monoterpene
**13**	*p*-cymene	0.11	3.36	0.10	0.52	monoterpene
**14**	Camphor	-	0.01	0.10	2.24	monoterpene
**15**	*p*-cymenol	-	0.17	0.03	0.03	other
**16**	Carvone	-	0.06	-	0.34	monoterpene
**17**	Carvacrol	-	0.22	-	/	monoterpene
**18**	β-caryophyllene	-	0.85	0.06	0.49	sesquiterpene
**19**	ar-curcumene	-	11.42	11.63	0.94	sesquiterpene
**20**	Caryophyllene oxide	-	0.69	-	0.43	sesquiterpene
**21**	1-(3-cyclopentylpropyl)-2,4-dimethylbenzene	-	1.47	-	-	other
**22**	Cedrenol	-	0.73	-	-	sesquiterpene
**23**	Curlone	-	7.59	-	-	sesquiterpene
**24**	1,8-cineol/eucalyptol	33.78	2.01	2.92	42.71	monoterpene
**25**	Camphene	-	-	5.84	4.58	monoterpene
**26**	Citronellol	-	-	0.07	-	monoterpene
**27**	Citronellyl acetate	-	-	0.17	-	monoterpene
**28**	α-copaene	-	-	0.21	0.05	sesquiterpene
**29**	δ-cadinene	-	-	0.19	0.38	sesquiterpene
**30**	α-calacorene	-	-	-	0.63	sesquiterpene
**31**	β-calacorene	-	-	-	0.08	sesquiterpene
**32**	Cubenol	-	-	-	1.26	sesquiterpene
**33**	Cadalene	-	-	-	0.74	sesquiterpene
**34**	Dimethyl styrene	-	0.05	-	-	other
**35**	4,4-dimethyl-3,4-dihydro coumarin	-	0.63	-	-	other
**36**	Ethyl dihydrocinnamate	-	-	-	0.10	other
**37**	β-elemene	0.11	-	0.36	-	sesquiterpene
**38**	δ-elemene	-	-	0.03	-	sesquiterpene
**39**	α-farnesene	-	-	2.26	-	sesquiterpene
**40**	endo-fenchol	-	-	-	0.03	monoterpene
**41**	exo-fenchol	-	-	-	0.12	monoterpene
**42**	Fenchyl acetate	-	-	-	0.55	monoterpene
**43**	D-germacrene	-	-	2.79	1.89	sesquiterpene
**44**	Geranial	0.08	-	0.04	-	monoterpene
**45**	Geraniol	0.24	-	-	-	monoterpene
**46**	Geranyl acetate	0.23	-	1.23	-	monoterpene
**47**	α-humulene	-	0.26	-	0.17	sesquiterpene
**48**	2-heptanol	-	-	0.04	-	other
**49**	Isobutyl isovalerate	-	-	-	0.08	other
**50**	Isoamyl isovalerate	-	-	-	0.04	other
**51**	Isoamyl-2-methylbutyrate	-	-	-	0.06	other
**52**	Isoborneol	-	0.01	1.42	0.60	monoterpene
**53**	Isobornyl acetate	-	-	0.58	0.22	monoterpene
**54**	Isobutyl benzoate	-	-	-	0.21	other
**55**	Limonene	2.32	0.15	7.90	2.76	monoterpene
**56**	Linalool	2.72	0.02	0.41	0.41	monoterpene
**57**	Linalyl acetate	0.67	-	-	-	monoterpene
**58**	Myrcene	0.78	0.05	0.43	0.20	monoterpene
**59**	6-methyl-5-hepten-2-one	-	0.01	0.13	0.41	other
**60**	Methyl isovalerate	-	-	-	0.05	other
**61**	Neral	-	-	0.36	-	monoterpene
**62**	Neryl acetate	0.08	-	-	-	monoterpene
**63**	Ocimene	0.06	-	-	-	monoterpene
**64**	α-pinene	0.98	0.20	1.83	3.56	monoterpene
**65**	β-pinene	0.26	0.03	0.15	3.85	monoterpene
**66**	Phenethyl isobutyrate	-	-	-	0.56	other
**67**	Phenethyl isovalerate	-	-	-	0.44	other
**68**	α-phellandrene	-	1.33	0.40	-	monoterpene
**69**	α-selinene	-	-	-	0.72	sesquiterpene
**70**	β-selinene	-	-	0.38	0.49	sesquiterpene
**71**	Sabinene	1.82	0.01	0.08	-	monoterpene
**72**	β-sesquiphellandrene	-	10.44	10.08	-	sesquiterpene
**73**	Sabinyl acetate	-	0.20	-	-	monoterpene
**74**	α-thujene	0.14	0.02	0.01	0.07	monoterpene
**75**	α-terpinene	0.36	0.03	0.03	0.25	monoterpene
**76**	γ-terpinene	0.61	0.03	0.04	0.31	monoterpene
**77**	Terpinolene	0.32	0.26	0.14	0.20	monoterpene
**78**	4-terpineol	1.85	0.08	0.21	2.29	monoterpene
**79**	α-terpineol	2.98	0.20	0.69	11.11	monoterpene
**80**	α-terpinyl acetate	42.65	0.46	0.21	0.31	monoterpene
**81**	ar-turmerone	-	12.28	-	-	sesquiterpene
**82**	β-turmerone	-	25.77	-	-	sesquiterpene
**83**	Tricyclene	-	-	0.12	0.11	monoterpene
**84**	Thymol	-	0.48	-	-	monoterpene
**85**	2-undecanone	-	-	0.38	-	other
**86**	Valencene	-	-	-	0.35	sesquiterpene
**87**	α-zingiberene	-	5.13	22.69	-	sesquiterpene
**Total monoterpenes (%)**		**93.04**	**9.26**	**25.52**	**77.40**	
**Total sesquiterpenes (%)**		**0.11**	**78.29**	**58.42**	**9.22**	
**Other compounds (%)**		**-**	**2.48**	**0.58**	**3.07**	
**Total compounds identified (%)**		**93.15**	**90.03**	**84.52**	**89.69**	

**Table 2:** Information of the analyzed plant material.

Common Name	Latin Name	Plant Part	Origin
Cardamom	*Elettaria cardamomum* L. Maton	fruits	Guatemala
Turmeric	*Curcuma longa* L.	rhizome	India
Ginger	*Zingiber officinale* Roscoe	rhizome	Madagascar
Galangal	*Alpinia officinarum* Hance	rhizome	China

## Supplementary Materials

The following are available online at https://www.mdpi.com/2223-7747/10/3/501/s1, Figure S1: Typical GC-MS/MS chromatogram of cardamom (*Elettaria cardamomum* L. Maton) essential oil, Figure S2: Typical GC-MS/MS chromatogram of turmeric (*Curcuma longa* L.) essential oil, Figure S3: Typical GC-MS/MS chromatogram of ginger (*Zingiber officinale* Roscoe) essential oil, Figure S4: Typical GC-MS/MS chromatogram of galangal (*Alpinia officinarum* Hance) essential oil.
