# Supplementary material for: Comparative Study of Chemical Composition and Antioxidant Activity of Essential Oils and Crude Extracts of Four Characteristic Zingiberaceae Herbs
Source: Plants (Basel). 2021 Mar 8;10(3):501. doi: 10.3390/plants10030501 (PMC7999660; doi:10.3390/plants10030501)
Supplement: Supplementary file 1 [file plants-10-00501-s001.pdf]

plants

MDPI

Supplementary Material

Comparative Study of Chemical Composition and Antioxidant Activity of Essential Oils and Crude Extracts of Four Characteristic *Zingiberaceae* Herbs

Milena Ivanović, Kaja Makoter and Maša Islamčević Razboršek *

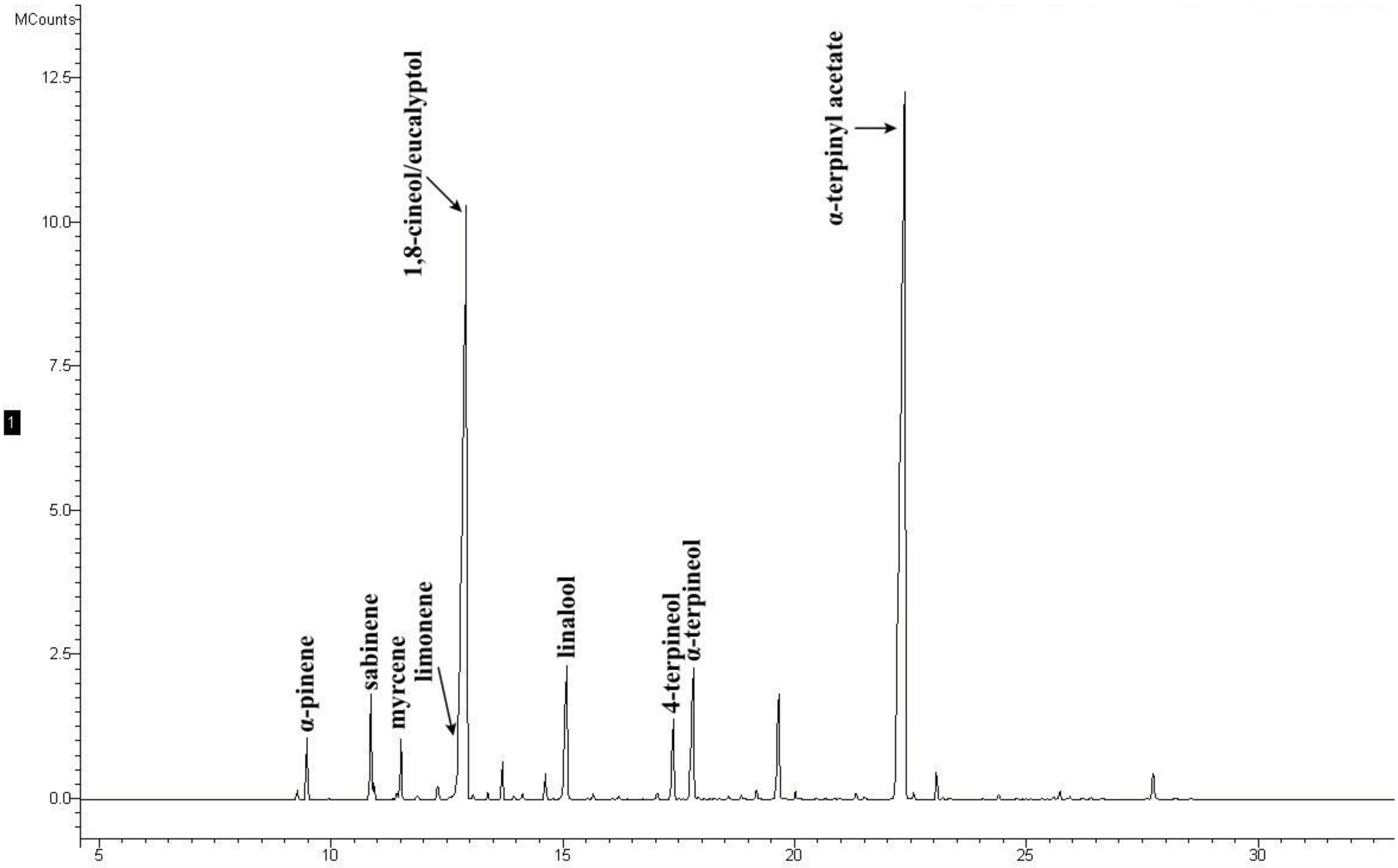

Figure S1. Typical GC-MS/MS chromatogram of cardamom (*Elettaria cardamomum* L. Maton) essential oil.

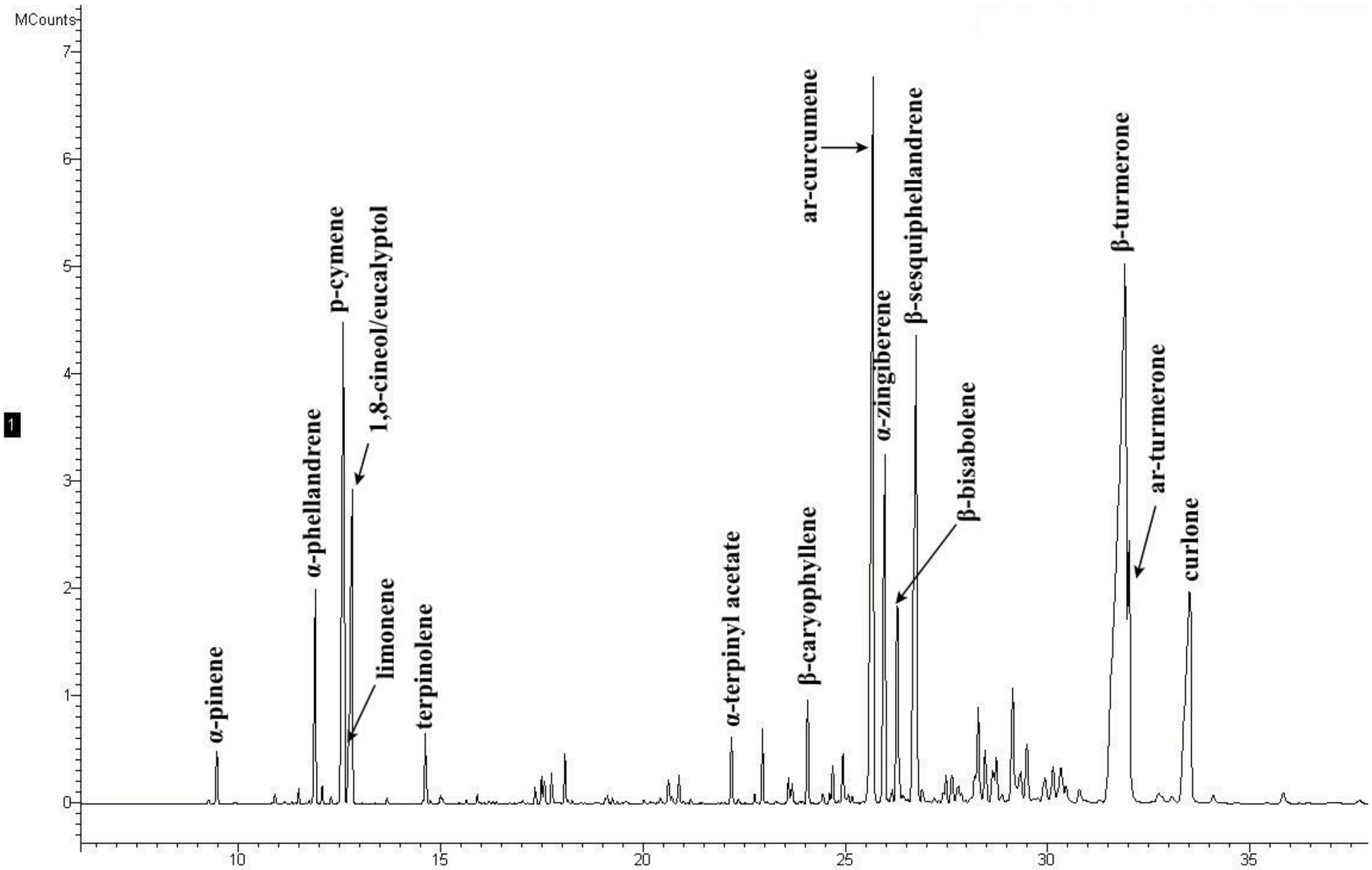

Figure S2. Typical GC-MS/MS chromatogram of turmeric (*Curcuma longa* L.) essential oil.

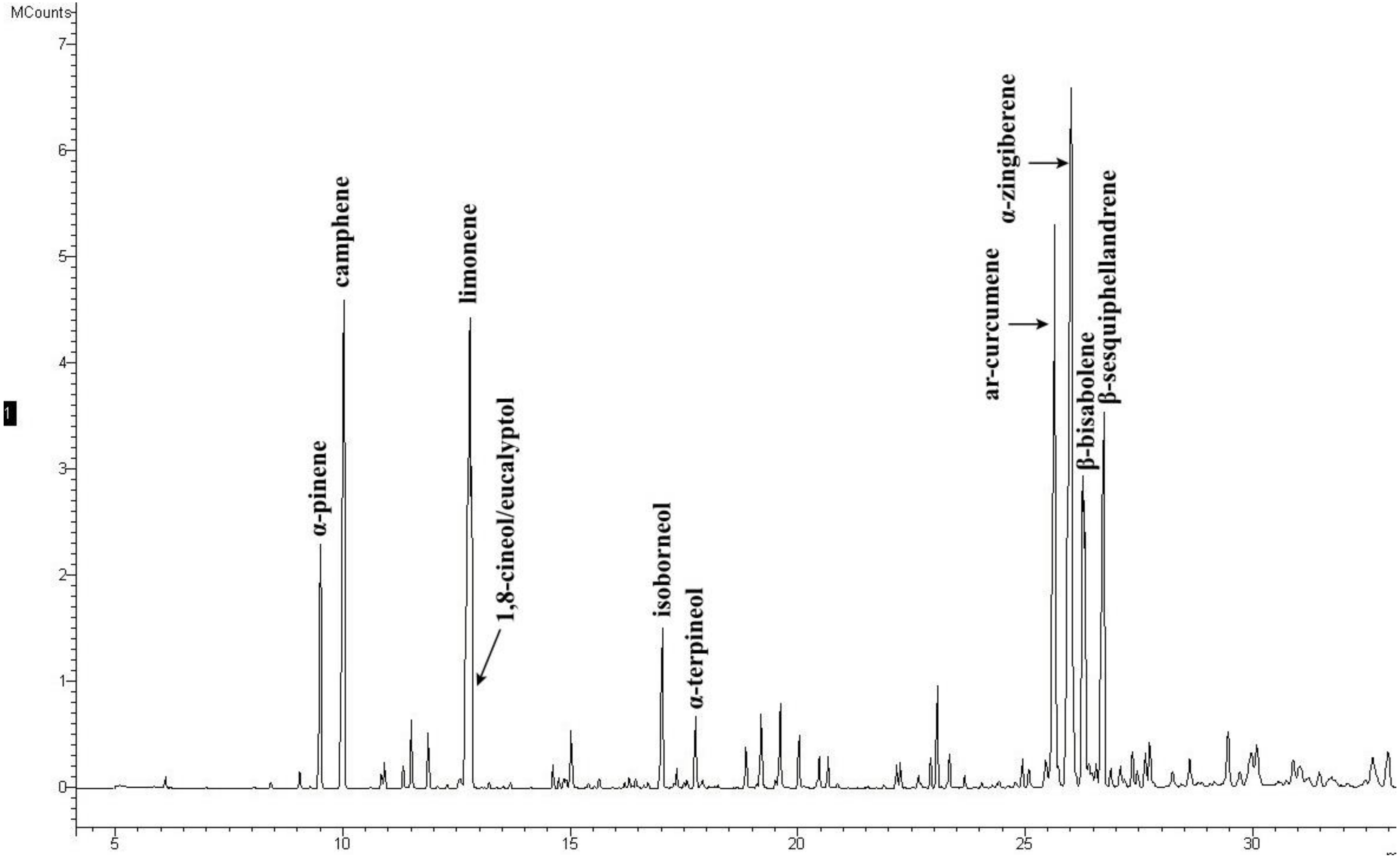

Figure S3. Typical GC-MS/MS chromatogram of ginger (*Zingiber officinale* Roscoe) essential oil.

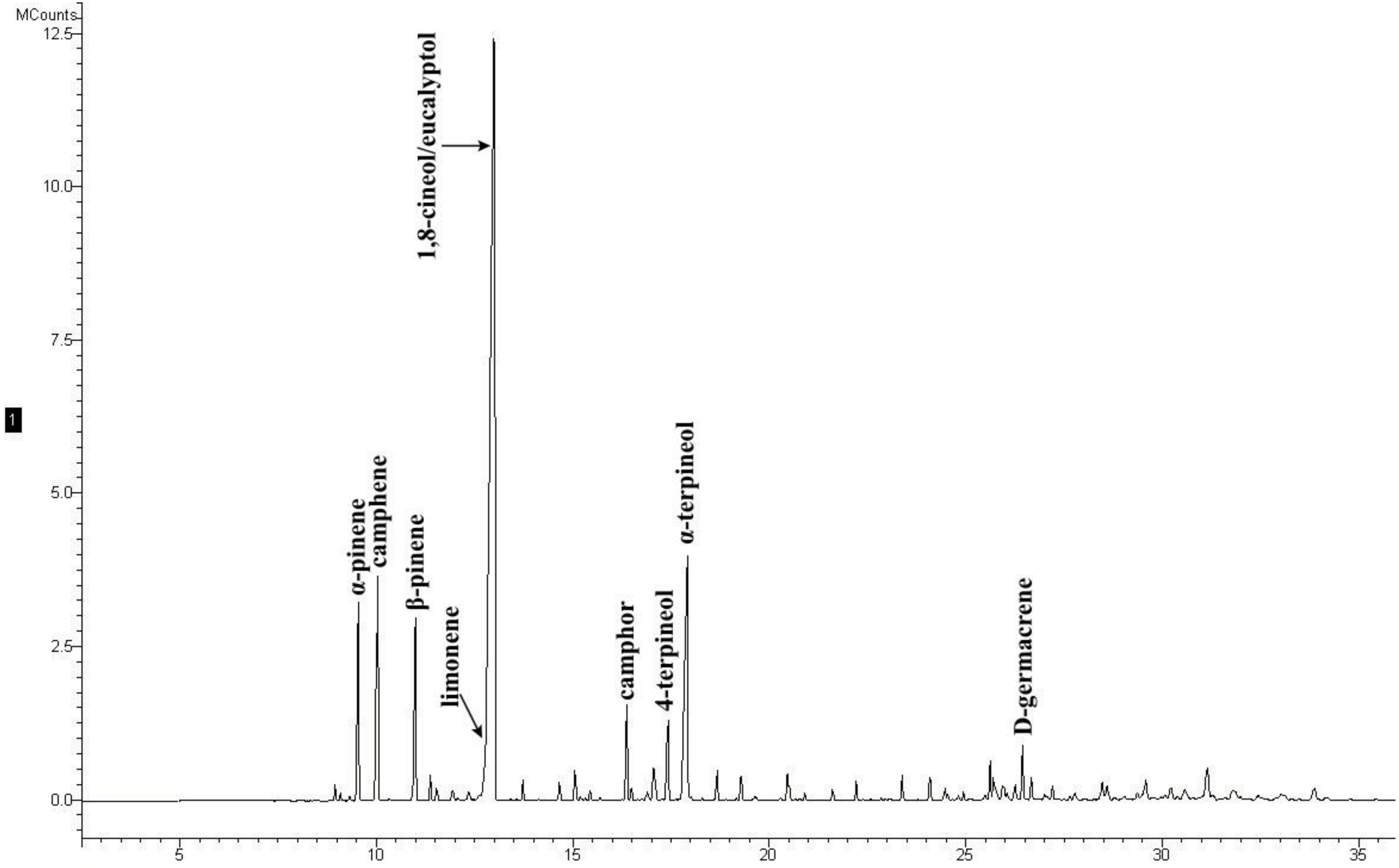

Figure S4. Typical GC-MS/MS chromatogram of galangal (*Alpinia officinarum* Hance) essential oil.
